# Editorial: Critical Perspectives on Replicability in Work/Organizational Psychology Research

**DOI:** 10.3389/fpsyg.2021.667479

**Published:** 2021-03-30

**Authors:** Giuseppe Scaratti, Yrjö Engeström, Silvio Ripamonti

**Affiliations:** ^1^Department of Human and Social Sciences, University of Bergamo, Bergamo, Italy; ^2^CRADLE, University of Helsinki, Helsinki, Finland; ^3^Department of Psychology, Catholic University of Milan, Milan, Italy

**Keywords:** critical approach, generativity, intervention, transformative research, engaged scholarship, epistemological/methodological implications

Thinking about Critical Perspectives on Replicability in Work/Organizational Psychology Research entails multiple and intertwined theoretical and operational stances and conveys challenges and suggestions for enhancing meaningful, interesting, and solid empirical research in the field of Work and Organizational Psychology (WOP).

On the one hand, the label of replicability refers to the prescriptive of a routinized approach to conducting research. On the other hand, it should be reconsidered in the light of the last decade's debates about changes in our understanding of linguistic, narrative, practical, and material aspects of work and organizations.

Organization studies is emerging as an applied and performative science (Alvesson, 2020), aimed at enhancing academic-practitioner collaboration, and going beyond narrowly circumscribed areas of study to pursue ground-breaking research. Much of the research in this field is context-specific and therefore requires a shift in the mode of knowledge production. The shift would be from traditional, linear knowledge production to knowledge production embedded in social processes of transformation, emphasizing material, historical, socio-linguistic, and relational conditions. How can researchers gain an understanding of organizations from within work activities, considering the social nature of organizations and the situated contextual conditions in which work takes place?

What is at stake is an epistemic awareness of a science immersed in the world. A science that, if oriented to the study of human action in its various manifestations, individual and collective, cannot evade responsibility for its uses, by bringing together truth and values, epistemic and ethical demands. Research needs to become capable of going beyond formulaic models and playing a role in configuring more influential and future-oriented approaches in the study of organizations and work.

In this issue, we display the growing importance of theoretically and methodologically ambitious intervention research which moves the focus from traditional replicability and statistical generalizability to generativity, understood as “locally initiated appropriate solutions that can lead to practical systemic transformation as well as to the development of novel theoretical and methodological research tools” (Sannino et al., 2016, p. 605). Authors on this issue acknowledge the importance of engaged scholarship (Van de Ven, 2018) and political and ethical commitment to the critical challenges of our time, including climate change, poverty, and the polarization of societies.

In their paper *Unmute the Organization through Serious Play*, Heldal et al. describe a research process structured around “serious play” and designed as a talk show, where researchers played parts, including that of a talk show host, and where questions pertaining to organizational life were discussed in depth, highlighting how generativity may emerge even if strong demands of classical replicability are not met.

In the paper *Will an Implementation of “Joy of Life in Nursing Homes” Have a Positive Effect for the Work Culture? A Comparison Between Two Norwegian Municipalities* (André et al.) address the Joy of Life Nursing Home (JoLNH) strategy, representing a resource-oriented health-promoting approach developed for enhancing the work culture in Norwegian nursing homes, valorizing repertoires of shared knowledge and common identification that evolve during the process.

The paper by Gobo, *Replicability: Politics and Poetics of Accountability, Validation, and Legitimation*, sheds light on a “situational approach” based on the idea that replicability works under certain organizational and socio-technic conditions, and that it is heavily influenced by the way that different stakeholders (scientists, technicians, participants, artifacts, and technologies) respond to them. The paper discusses epistemological implications related to going beyond the linear progression from theory to application, moving between the content of knowledge and the locally acknowledged use(s) of knowledge.

In their paper *From a Sociological Given Context to Changing Practice: Transforming Problematic Power Relations in Educational Organizations to Overcome Social Inequalitie*s, Lémonie et al. refer to the tradition of Cultural-Historical Activity Theory (CHAT), presenting the first steps in interventionist research that accompanied the implementation of public policy aimed at reducing social inequalities in access to educational success in France.

In the paper *Does Workplace Bullying Produce Employee Voice and Physical Health Issues? Testing the Mediating Role of Emotional Exhaustion* Liang addresses the bullying process, focusing on victims who experience a negative bullying environment. The paper addresses a critical challenge related to the spreading of problematic phenomena in workplaces, seeking ways to improve an organization's capability to deal with them.

The paper by Lusardi, *The Contingency of the Lifeworld in a World of Standards: Repertoires of Resignification in (Evidence-Based) Healthcare Organization*, describes an innovative method to approach and highlight tacit situated knowledge, developing ways for accompanying subjects to revisit and reflect on their in-use assumptions. The author's Science and Technology Study (STS) perspective, as a practice-based analysis of daily work, shows how interactions between human actors, technological artifacts, and organizational apparatus constitute repertoires of resignification for resolving the tension between medicine's universalistic aspirations and the unpredictable, situated nature of the lifeworld.

In their paper *Learning Platforms for Implementing Formative Interventions to Promote the Health and Safety of Workers in Brazil*, Lopes et al. describe the adoption of the formative intervention methodology of the Change Laboratory (CL) as an emblematic example of transformative intervention research. The paper examines the development of learning and training strategies for implementing formative interventions, drawing on the experiences of a research group focused on workers' health.

The last paper, *Uniqueness and Generalization in Organizational Psychology: Research as a Relational Practice*, by Scaratti and Ivaldi, argues for research as relational practice, highlighting the epistemological and methodological implications of conceiving Work and Organizational Psychology as an idiographic, situated, and transformative social science. Such research is aimed at developing forward-oriented sense making and enabling a participatory approach that conceives subjects as co-authors, negotiating with them the aims, methods, and mutual expectations of research.

## Author Contributions

GS supervised the whole process, while SR managed the reviewing process and YE provided the improvement of the quality of the contributions submitted. All authors contributed to the article and approved the submitted version.

## Conflict of Interest

The authors declare that the research was conducted in the absence of any commercial or financial relationships that could be construed as a potential conflict of interest.
